# Toward smart health monitoring: multimodal sensing and intelligent disease diagnosis in poultry and livestock

**DOI:** 10.1093/af/vfaf060

**Published:** 2026-01-09

**Authors:** Juncheng Ma, Xiao Yang, Yu Liu, Peiguang Xin, Qin Tong, Chao Liang, Ligen Yu, Aiqiao Liu, Chaoyuan Wang

**Affiliations:** Department of Agricultural Structure and Bioenvironmental Engineering, College of Water Resources and Civil Engineering, China Agricultural University, Beijing 100083, China; Key Laboratory of Agricultural Engineering in Structure and Environment, Ministry of Agriculture and Rural Affairs, Beijing 100083, China; Department of Agricultural Structure and Bioenvironmental Engineering, College of Water Resources and Civil Engineering, China Agricultural University, Beijing 100083, China; Key Laboratory of Agricultural Engineering in Structure and Environment, Ministry of Agriculture and Rural Affairs, Beijing 100083, China; Department of Agricultural Structure and Bioenvironmental Engineering, College of Water Resources and Civil Engineering, China Agricultural University, Beijing 100083, China; Key Laboratory of Agricultural Engineering in Structure and Environment, Ministry of Agriculture and Rural Affairs, Beijing 100083, China; Department of Agricultural Structure and Bioenvironmental Engineering, College of Water Resources and Civil Engineering, China Agricultural University, Beijing 100083, China; Key Laboratory of Agricultural Engineering in Structure and Environment, Ministry of Agriculture and Rural Affairs, Beijing 100083, China; Department of Agricultural Structure and Bioenvironmental Engineering, College of Water Resources and Civil Engineering, China Agricultural University, Beijing 100083, China; Key Laboratory of Agricultural Engineering in Structure and Environment, Ministry of Agriculture and Rural Affairs, Beijing 100083, China; Department of Agricultural Structure and Bioenvironmental Engineering, College of Water Resources and Civil Engineering, China Agricultural University, Beijing 100083, China; Key Laboratory of Agricultural Engineering in Structure and Environment, Ministry of Agriculture and Rural Affairs, Beijing 100083, China; Research Center of Information Technology, Beijing Academy of Agriculture and Forestry Sciences, Beijing 100097, China; Beijing WOD-Botron Information Technology Co., Ltd, Beijing 101200, China; Department of Agricultural Structure and Bioenvironmental Engineering, College of Water Resources and Civil Engineering, China Agricultural University, Beijing 100083, China; Key Laboratory of Agricultural Engineering in Structure and Environment, Ministry of Agriculture and Rural Affairs, Beijing 100083, China

**Keywords:** artificial intelligence, health monitoring, intelligent disease diagnosis, multimodal fusion, poultry and livestock

Implications

- Multimodal sensing provides valuable data and knowledge for intelligent disease diagnosis.
- Intelligent disease diagnosis is evolving from text-based understanding toward multimodal fusion and knowledge-driven reasoning.
- Future efforts should prioritize devices for definite disease diagnosis and diagnostic frameworks integrating prescription generation for intelligent decision support.

## Introduction

Health management of livestock and poultry production is critical for ensuring food safety, animal welfare, and production sustainability. Farm scales expanded rapidly in China over the last two decades. For example, the capacity of a single laying hen house increased from thousands to hundreds of thousands. Large-scale farm typically refers to an annual slaughtered volume of >100,000 birds or >50,000 pigs. As such, the prevention and control of animal diseases have become increasingly complex. For instance, African Swine Fever (ASF), which can have a mortality rate of up to 100%, exemplifies the devastating impact of animal epidemics. The largest ASF outbreak in China in 2018 led to the death of 43.46 million pigs, causing an estimated US$14.5 billion in indirect economic losses (You et al., 2021). However, many poultry and livestock farms often suffer from limited diagnostic infrastructures and professional expertise. Veterinarians are usually responsible for multiple farms, which may increase the risk of cross-infection and substantial economic loss. In addition, the shortage of licensed veterinarians further increases the difficulty of timely health management (Yang et al., 2025). Recent advances in artificial intelligence (AI) and smart sensing offer promising solutions and enable automated health monitoring and intelligent diagnostic systems, which marks a crucial step toward the digital transformation of livestock health management.

The evolution of intelligent disease diagnosis has progressed through three milestones, ie, the Database Matching System (DMS), the Expert System (ES), and the Large Language Model (LLM). The DMS, originating in the 1960s, marked the beginning of intelligent diagnosis. It functions by calculating similarity scores through keyword matching and weighted symptom scoring to suggest suspected diseases. While effective for common, well-documented diseases, the DMS accuracy declines in cases involving atypical symptoms, co-infections, or emerging diseases (Houe et al., 2011). Today, database-driven systems are incorporated into cloud-based diagnostic platforms and veterinary knowledge graphs, continuing to serve as useful tools for rapid, simple, and low-cost screening on small- and medium-scale farms. Building upon the DMS, the ES are developed to emulate specialists' reasoning processes through if-then rules. The MYCIN system, introduced by Stanford University in the 1970s for diagnosing human infectious diseases, exemplified this approach (Saeidnia and Nilashi, 2025). In animal management, for example, the ES has been used to diagnose diseases such as blue ear in pigs, Newcastle disease in poultry, respiratory disease in dairy cattle. While these systems provide interpretable reasoning chains, they lack self-learning capability and adaptability, making them less effective for complex or uncertain scenarios. By the 2020s, the field has transitioned from rule-based reasoning to data-driven learning. The advent of LLMs such as Generative Pre-trained Transformer (GPT) and Pathways Language Model (PaLM) leverages massive datasets and deep learning architectures to autonomously acquire knowledge and detect subtle correlations across modalities (Jin et al., 2025). Although LLMs exhibited broad coverage and strong cross-domain integration ability, their “black-box” nature limits interpretability and complicates the validation of knowledge sources.

Effective intelligent diagnosis in poultry and livestock health relies fundamentally on the reliability of sensed data. Traditional approaches have often relied on single-modality data due to its simplicity, cost-effectiveness, and ease of deployment. While these approaches can provide meaningful insights under controlled conditions, and form the basis of early database matching and expert system-based diagnostics, they suffer from limitations in robustness and accuracy due to environmental interference, noise, or biological variability. For instance, infrared thermal imaging may misinterpret surface heat changes caused by airflow as fever, and RGB cameras may struggle to estimate body weight under poor lighting or occlusion (Liu et al., 2023).

To overcome these constraints, recent research emphasizes multimodal sensing as a powerful approach by integrating heterogeneous data sources. The advantages of multimodal sensing over single-modality approaches are threefold. First, redundancy increases resilience, allowing one modality to compensate when another is compromised. Second, complementarity allows each sensor to contribute unique insights to the same health indicator. Third, robustness is achieved through integrating multiple data streams, enabling diagnostic models to generalize across varying farm environments and animal populations.

**Figure 1.**
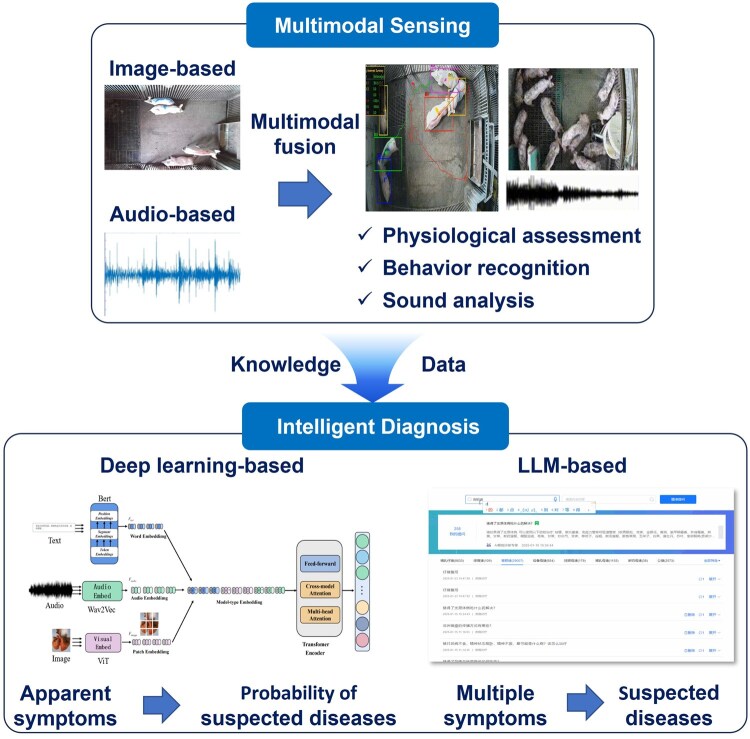
Multimodal sensing and intelligent disease diagnosis in poultry and livestock. The deep-learning based disease diagnosis model was adapted from Yang (2025). LLM donated Large Language Model.

Therefore, this review will focus on the applications of multimodal fusion in health information perception of farm animals, summarizing recent achievements in physiological condition assessment, behavior recognition and abnormal sound analysis. Afterwards, multimodal fusion-based technologies of diagnosis models will be introduced (Figure 1). Finally, the challenges and limitations of intelligent diagnosis of livestock and poultry, along with future perspectives, will be discussed to provide insights into the revolution of farm animal health management.

## Multimodal Sensing for Animal Health Monitoring

### General pipelines of multimodal fusion

**Figure 2.**
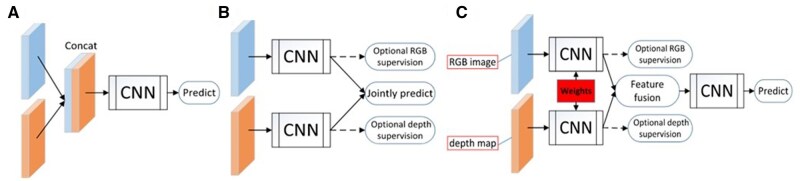
Multimodal fusion framework, taking RGB-D fusion as an example (Fu et al., 2022). (a) Early-fusion. (b) Late-fusion. (c) Middle-fusion. CNN donated Convolutional Neural Network, and Concat donated concatenate‌.

Multimodal sensing enhances animal health monitoring by automatic physiological condition assessment, behavior recognition and abnormal sound analysis. As sensors become increasingly common in livestock and poultry farms, multiple data modalities can be collected simultaneously. Typically, information contained in different data modalities varies and is specific to particular scenarios. However, in farm environments, the application scenario may pose challenges due to many factors, such as changing illumination, background noise, and limited image resolutions, making it unreliable to rely on a single data modality for decision-making (Yin et al., 2023; Ma et al., 2025). Multi-modality fusion integrates multiple sources of data, such as environmental factors, images, and audio, providing a more comprehensive understanding of the environment in livestock and poultry farms compared with a single data modality and enabling more accurate results in challenging farming environments (Kalamkar, 2023; Tang et al., 2023). Therefore, it is necessary to employ multimodal fusion to enhance the animal health monitoring and disease diagnosis. According to the fusion level, the deep learning-based multimodal data fusion can be divided into data-level fusion, feature-level fusion, and decision-level fusion (Tang et al., 2023), as shown in Figure 2.

### Image-based multimodal sensing

**Figure 3.**
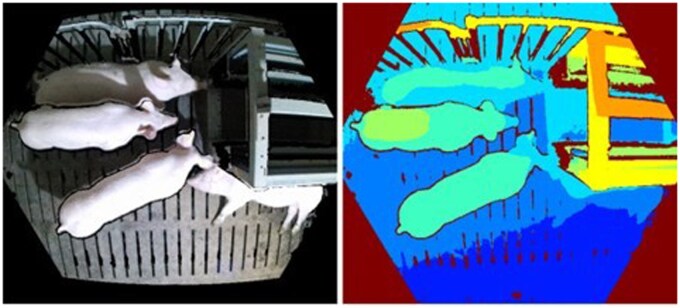
Paired RGB (left) and Depth (right) images (He et al., 2023). A Microsoft Azure Kinect DK camera was adopted to simultaneously collect the paired RGB-D images. The camera was horizontal to the ground at a height of 2.3 m above the feeding passageway.

**Figure 4.**
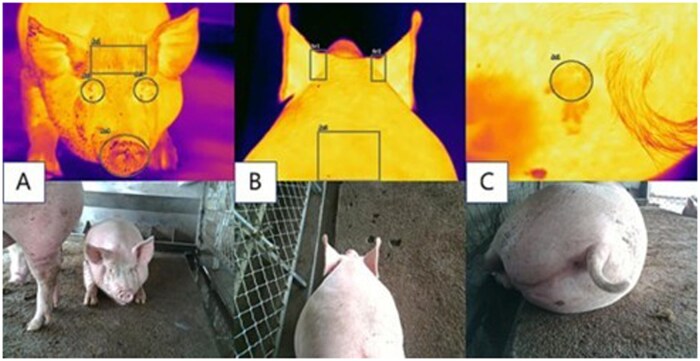
Paired thermal (top) and RGB (bottom) images of six different regions on pig body surface (Xie et al., 2023). A: forehead, eyes, and nose. B: ear root, and back. C: anus. The thermal and visible light images could be obtained at the same time by using an infrared thermal imaging camera.

Among the sensors used in livestock and poultry farms, cameras are one of the most widely used devices significantly contributing to the non-contact perception and visible ­livestock management by computer vision (Li et al., 2022a). Generally, image modalities used in computer vision for livestock ­management include Red-Green-Blue (RGB), depth (Figure 3), and thermal (Figure 4) images. Given the low cost and high accessibility, RGB images are one of the most common image modalities, offering rich color and texture information with a high spatial resolution (Ma et al., 2023). As a result, RGB images are widely adopted in various applications in livestock and poultry farms. Nevertheless, the long-existing challenges inherent in complicated farming conditions, such as changing illumination and clutter backgrounds (Lamping et al., 2022; Li et al., 2022a), need to be addressed before the RGB image-based methods can achieve improved performance (Liu et al., 2022).

Recent advancements in imaging technology and hardware have made depth images readily available through consumer-level RGB-D cameras, effectively complementing the RGB images. In a depth image, each pixel represents the distance from the camera to the corresponding object, which favors the 3D reconstruction of animals (Li et al., 2022b). Furthermore, depth images provide shape information, which helps extract animal objects from clutter backgrounds (Xu et al., 2022). In most cases, depth images are combined with RGB images since RGB images can remedy the defects of coarse resolution and details (Liu et al., 2022; He et al., 2023). Typically, RGB images have high spatial resolution along with rich color and texture information (Ma et al., 2023), whereas they are highly susceptible to illumination variations and lack structure information (Liu et al., 2022; Xu et al., 2022). In contrast, depth images are robust to illumination changes and provide substantial shape and 3D structure details (Xu et al., 2022; He et al., 2023), while they lack fine object details (Liu et al., 2022; He et al., 2023). The RGB-D fusion leverages the complementary strengths of both modalities, enabling a more comprehensive understanding of animal health and improving the estimation of physiological traits, such as pig body weight (He et al., 2023), body size (Li et al., 2022b), posture (Xu et al., 2023) and appearance (Lamping et al., 2022).

Thermal images can also serve as a complementary modality, especially under low illumination and complex background conditions due to the robustness against lighting variations (Wu et al., 2023). Thermal images measure the animal surface temperature (Cai et al., 2023; Xie et al., 2023), which is a key indicator of animal health and welfare (Cai et al., 2023). Consequently, thermal images are widely used in livestock and poultry farms to detect animal temperature and diseases (Xie et al., 2023). Additionally, thermal images can also aid in extracting animals from clutter backgrounds, as animals typically have higher temperatures than objects in the backgrounds (Zhong and Yang, 2022). Although thermal images have shown great potential in livestock and poultry farms, the high cost of the thermal infrared camera significantly hinders their use. Besides, image alignment is also required for fusion between RGB and thermal images. Notably, in applications where thermal images are used to measure animal surface temperatures, the accuracy may be influenced by the ambient temperature and humidity, measuring distance and angle, and emissivity of measuring parts (Xie et al., 2023).

### Audio-based multimodal sensing

**Figure 5.**
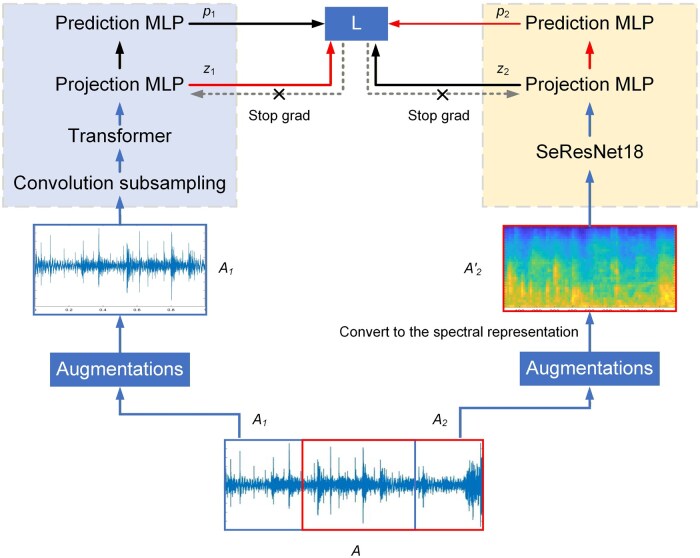
Structure of the multimodal pig audio representation and fusion framework (Ma et al., 2025). A was a given audio clip, $A_{1}$ and $A_{2}$ were two randomly cropped segments, $A_{2}^{\prime }$ was the spectral representation of $A_{2}$. $z_{1}$ and $p_{1}$ were the output of $A_{1}$, $z_{2}$ and $p_{2}$ were the output of $A_{2}^{\prime }$, Stop grad indicated the stop gradient operation and MLP donated multilayer perceptron.

In addition to camera, acoustic sensors offer a valuable means of linking animal vocalizations to health, welfare, and environmental impact (Ma et al., 2025). In livestock and poultry farms, acoustic technologies have been developed to detect a multitude of traits and behaviors, such as coughing (Shen et al., 2022; Ma et al., 2025), body weight (Fontana et al., 2017), diseases (Cuan et al., 2020), and feeding behavior (Liao et al., 2022). Given the complicated acoustic environment in livestock and poultry farms (Shen et al., 2022; Ma et al., 2025), the sensing models relying solely on acoustic features may not achieve satisfactory accuracy. Instead, fusing the acoustic features with other powerful features can improve the detection accuracy (Shen et al., 2022; Ma et al., 2025), as shown in Figure 5. Although promising results have been reported (Shen et al., 2022; Ma et al., 2025), it is worth noting that the features mentioned above are derived from the same data as the acoustic features. Therefore, the fusion of acoustic data and other data modalities is yet to be explored.

## Intelligent Disease Diagnosis in Poultry and Livestock

### Deep learning-based intelligent disease diagnosis

Traditional diagnostic methods relying on manual observation and laboratory testing often suffer from low efficiency, high costs, and limited accuracy. With the advancement in natural language processing, computer vision, audio signal analysis, and knowledge graph, researchers (Hoang et al., 2023; Mustapoevich et al., 2023; Wang et al., 2024; Yu et al., 2024; Li et al., 2025) have developed intelligent diagnostic models that integrate multimodal and knowledge-driven approaches to improve accuracy, interpretability, and responding speed.

Text-based intelligent diagnosis models form the foundation of this field. Yu et al. (2024) developed a Bidirectional encoder representation from transformers-Bidirectional long short-term memory network-Conditional random field (BERT-BiLSTM-CRF) model that effectively handled sparse domain texts with an F1 Score (a widely used performance metric in machine learning) of 96.38%. Wang et al. (2024) integrated BERT semantic vectors with knowledge embeddings to analyze 11,401 laying-hen cases, significantly improving diagnostic accuracy in complex textual contexts. Similar knowledge-driven text learning methods have also shown robustness in scenes where the number of training samples was limited (Wang et al., 2023). Although these models offer strong semantic comprehension, they are still limited by issues such as data imbalance, text ambiguity, and poor generalization (Yang et al., 2025).

To overcome the limitations of unimodal diagnosis model, multimodal diagnosis models that integrate linguistic and visual information provide a feasible solution. Li et al. (2025) designed an RGB-guided depth-image restoration network for dairy-cow monitoring, enhancing image completeness and data quality. Yang et al. (2025) developed a multimodal swine disease model that integrates CNN and BERT through cross-modal attention, improving Area Under Curve (AUC) by 6.8%. The Text-guided fusion network for swine diagnosis (TGFN-SD) further advanced this approach by dynamically regulating visual feature extraction through semantic guidance, achieving a macro-F1 score of 94.4%. Although challenges remain in data labeling, multimodal synchronization, and computational cost, these studies demonstrate that integrating image and text data enhances robustness and reduces diagnostic ambiguity.

**Figure 6.**
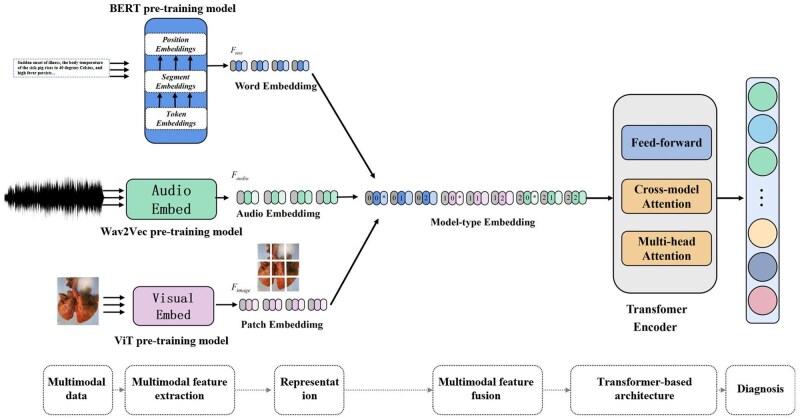
Diagnostic model structure of Yang (2025). ViT donated vision Transformer.

The inclusion of audio signals, such as respiration, coughing, and vocalization, provides additional information for real-time disease diagnosis. Mustapoevich et al. (2023) applied fuzzy-logic reasoning to cattle diseases, demonstrating the potential of non-visual data in identifying metabolic disorders. Yang (2025) incorporated cough and breathing sounds into a tri-modal model for pigs and used a CNN-Transformer architecture to fuse sound, image, and text features (Figure 6), achieving an AUC of 0.943 in respiratory disease detection. While these models enable early diagnosis, they still require standardized data acquisition and noise-robust preprocessing to ensure reliability.

**Figure 7.**
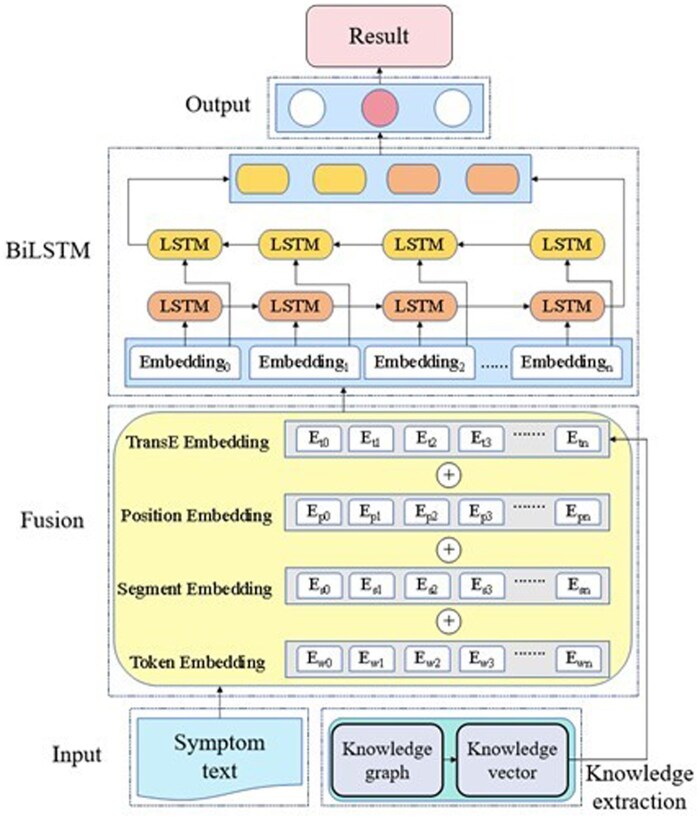
Diagnostic model structure of BERT-LHDKG (Wang et al., 2024).

Knowledge graph-based models incorporate structured reasoning and domain knowledge into intelligent diagnosis. Wang et al. (2024) proposed the bidirectional encoder representation from transformers-laying hens disease knowledge graph (BERT-LHDKG) model (Figure 7) for laying-hen disease diagnosis. The model integrated knowledge triplets (h, r, t) into BERT’s embedding layers and enhances feature extraction with BiLSTM, achieving a macro-F1 score of 94.01%, and outperforming ERNIE-BiLSTM by 2.19%. Similarly, Hoang et al. (2023) introduced the LiteralKG model, which incorporated attribute-aware embeddings for companion-animal disease reasoning. These frameworks establish explainable links between textual data and biological knowledge, allowing cross-species disease reasoning and improved interpretability.

Overall, research progress shows a clear trajectory from text-based understanding toward multimodal fusion and knowledge-enhanced reasoning. Despite significant advances, challenges remain in dataset scale, synchronization across modalities, and noise interference. Building large-scale multimodal datasets and embedding causal reasoning into diagnostic systems will be crucial for achieving interpretable and reliable intelligent diagnosis for livestock and poultry diseases.

### LLM-based intelligent disease diagnosis

Recent advances in LLMs such as GPT (Radford et al., 2018) and LLaMA (Touvron et al., 2023) have led to remarkable ­progress in reasoning, contextual understanding, and multimodal integration. As a result, livestock and poultry disease diagnosis is shifting from rule-based systems to data-driven, knowledge-augmented AI frameworks. However, adapting LLMs from general conversational contexts to veterinary and livestock health domains presents significant challenges, including limited domain-specific data, complex symptom overlaps across multiple species, and the need for interpretable and trustworthy predictions.

Early explorations by Coleman and Moore (2024) marked the first attempts to evaluate general large language models (LLMs), such as ChatGPT-3.5 and GPT-4, within a veterinary context. The results showed that GPT-4 achieved a mean accuracy of 77% on veterinary examination questions, outperforming GPT-3.5 (55%), yet still lagging behind veterinary students (86%). This result highlighted a crucial limitation that general LLMs lack the domain precision and contextual reliability required for professional veterinary diagnosis despite their strong linguistic and general reasoning abilities. With the advancement of agent-based technologies, research has shifted from improving individual model reasoning toward developing collaborative, workflow-oriented architectures. Frameworks such as MetaGPT (Hong et al., 2024) exemplify this trend. Following this direction, Mairittha et al. (2025) proposed a multi-agent AI framework for swine disease detection, integrating query classification, disease-specific reasoning, and retrieval-augmented generation (RAG). Dedicated disease agents were engaged in multi-stage questioning, ie, general, external, and specific symptom collection, before reaching a consensus through confidence-weighted decision fusion. This design enables adaptive, multi-turn diagnostic dialogue, simulating virtual veterinary consultations and achieving diagnostic accuracy above 90%. The proposed “confidence-weighted fusion” mechanism marks a shift from single-step inference to dynamic cooperative reasoning, where multiple agents collaborate to generate evidence-based conclusions. Recognizing that many livestock diseases manifest through visual and textual cues, subsequent research emphasized multimodal intelligence. Wen et al. (2025) proposed an intelligent diagnostic framework for porcine gastrointestinal infectious diseases, integrating textual symptom analysis with anatomical image interpretation. The system employed a multi-scale TextCNN for textual features, and an improved Mask R-CNN for lesion segmentation. Then, the model ensembled classifiers such as Random Forest and XGBoost for decision fusion. Remarkably, ChatGPT was leveraged to expand textual datasets sixfold, enriching linguistic variability and improving model generalization. The multimodal fusion achieved an overall accuracy of 87.6%, surpassing both text-only and image-only models. The results demonstrating that cross-modal feature integration combined with LLM-based data augmentation can substantially enhance diagnostic robustness in veterinary applications.

**Figure 8.**
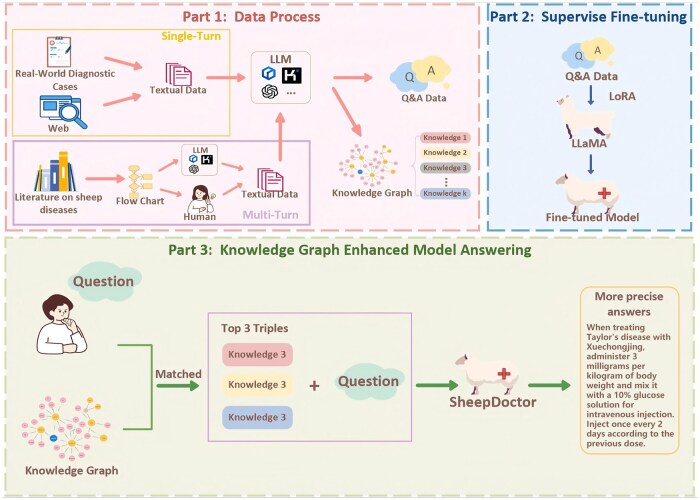
The framework of SheepDoctor (Xiong et al., 2025). The current version of the figure was a translation, as the original model answering output of SheepDoctor was in Chinese.

Most recently, research has advanced toward knowledge-grounded and explainable large models. Xiong et al. (2025) developed the SheepDoctor (Figure 8), the first knowledge-graph-enhanced large language model for sheep disease diagnosis. Built upon LLaMA2–13B with LoRA fine-tuning, SheepDoctor integrated a structured veterinary knowledge graph containing over 1,900 triples of disease–symptom–treatment relationships. Each user query was semantically aligned with relevant knowledge triples using BERT-based retrieval before being processed by the LLM, enabling contextualized and evidence-supported responses. The system demonstrated superior performance to general models such as GPT-4o and Kimi, and maintained high generalization when confronted with unseen diseases (F1 = 78.15%). Beyond accuracy, SheepDoctor introduced traceable, interpretable diagnostic reasoning, bridging the gap between statistical generation and symbolic veterinary knowledge.

The above studies highlight a clear trend of evolution from general LLM evaluation to domain-specialized, knowledge-augmented intelligence. The field has progressed from single-model inference to multi-agent reasoning, from unimodal to multimodal learning, and toward interpretable, knowledge-grounded frameworks. Collectively, these advances will transform veterinary and livestock health management from text understanding to holistic, multimodal, and knowledge-driven intelligent disease diagnosis.

## Challenges and Limitations

**Figure 9.**
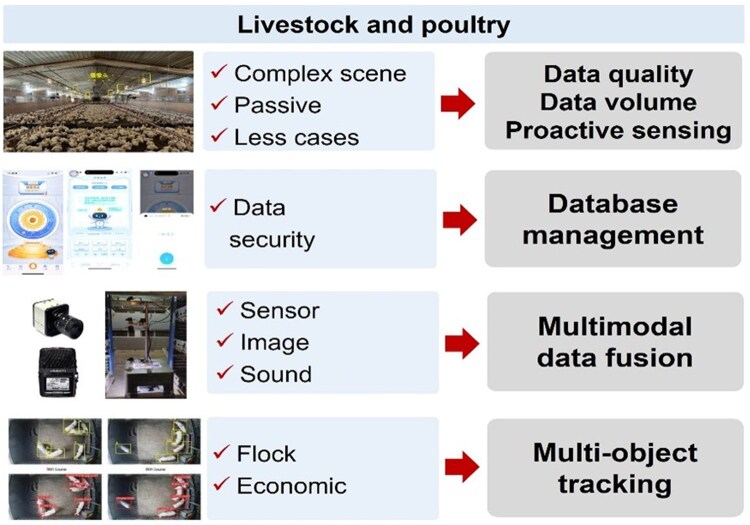
Challenges of intelligent diagnosis of poultry and livestock diseases.

Despite remarkable progress, intelligent diagnosis of poultry and livestock diseases still faces several critical challenges (Figure 9). First, the collection and annotation of large-scale, multimodal, and high-quality datasets remain a major bottleneck (Ma et al., 2025). Health-related data often require expert interpretation, which is labor-intensive, time-consuming, and prone to be inconsistent (Esteva et al., 2019; Ma et al., 2025). Abnormal or diseased samples are particularly scarce due to the low incidence of outbreaks and the rapid culling strategies typically adopted in commercial production systems. Moreover, disease records are rarely made publicly available owing to concerns over data privacy and biosecurity, limiting data sharing and model training. Second, high stocking densities are common in modern intensive livestock and poultry production systems, making precise individual health monitoring, such as physiological condition assessment, behavior recognition, and abnormal sound analysis, extremely challenging. Finally, advanced sensing technologies such as infrared imaging, depth cameras can be prohibitively expensive for small- and medium-scale farms, reducing the feasibility of large-scale applications. Besides, the generalization and cross-farm adaptability of the perception and diagnostic models remain challenging. Differences in animal breeds, management systems, and environmental conditions introduce both intra- and inter-species variability, undermining model robustness and transferability (Ma et al., 2025).

## Future Perspectives

The development of cost-efficient and non-invasive sensing technologies is essential for the large-scale deployment of intelligent diagnostic systems. The integration of edge computing and energy-efficient IoT devices can further reduce operational costs while enabling real-time, on-farm diagnostics that are accessible to small- and medium-sized producers. In addition, since many diseases share similar physiological or behavioral manifestations, transforming abnormal and suspected cases into confirmed diagnoses remains a major challenge and a critical milestone for further research. For example, intelligent interpretation of pathological images and the development of rapid pathogen detection devices may help with disease confirmation. Beyond detection, incorporating prescription generation into diagnostic frameworks represents the next step toward intelligent decision support, allowing systems to not only identify diseases but also recommend targeted treatments and management strategies. This evolution will transform smart livestock health management from a reactive to a proactive, autonomous, and precision-oriented paradigm.

## Conclusion

Advancements in AI are reshaping poultry and livestock health management. Given the complex farming environments, multimodal sensing, which holds significant advantages of robustness and high accuracy compared with single data modalities, has shown significant potential in animal physiological condition assessment, behavior recognition and abnormal sound analysis, providing valuable data and knowledge for intelligent disease diagnosis. With the collaboration of LLMs and edge intelligence, intelligent disease diagnosis is entering a new era characterized by multimodal fusion and knowledge-enhanced reasoning, enabling real-time monitoring and precision management for animal health. However, data scarcity and cross-farm variability persist, necessitating more robust intelligent diagnosis models. Future efforts should prioritize devices for definite disease diagnosis and diagnostic frameworks integrating prescription generation for intelligent decision support.

## Author Biographies

**Juncheng Ma** received a Ph.D. in Agricultural Engineering from China Agricultural University in 2016. He worked as an associate researcher with Chinese Academy of Agricultural Science from 2016 to 2023. Currently, he works as a researcher with College of Water Resources and Civil Engineering, China Agricultural University. He is a recipient of Young Elite Scientists Sponsorship Program by China Association for Science and Technology. He has extensive experience in intelligent perception of multimodal information for livestock and poultry, and intelligent disease diagnosis, developing the intelligent perception approach for behavior and physiological traits in complex farm environments. He has published more than 30 peer-reviewed papers.

**Xiao Yang** is an Assistant Professor in the Department of Agricultural Structure and Bioenvironmental Engineering at the College of Water Resources and Civil Engineering, China Agricultural University. She received her Ph.D. from the Department of Animal Science at University of Tennessee. Her research focuses on the development of precision livestock farming technologies and intelligent systems for modern animal production. Her major research areas include smart sensor–based monitoring of poultry behavior, machine-vision–enabled health assessment, automatic welfare evaluation, and early-warning systems for disease detection. Dr Yang has served as the principal investigator (PI) or co-PI on several competitive research projects, including grants from the National Natural Science Foundation of China, China Postdoctoral Science Foundation, etc. She has published more than 20 peer-reviewed research articles and conference proceedings, and contributed to the editing of four books chapters. She also involved on the development of national and industry standards on livestock production of China. Her work aims to advance data-driven, automated, and sustainable livestock farming by integrating sensing technologies, artificial intelligence, and engineering solutions to improve animal health, welfare, and production efficiency.

**Liu Yu** earned a Ph.D. in agricultural bio-environment and energy engineering from the China Agricultural University in 2022. She once conducted postdoctoral research in agricultural engineering at the Beijing Academy of Agriculture and Forestry Sciences. Since 2024, she has been an associate professor in agricultural bio-environment and building engineering department of China Agricultural University, mainly engaged in research on precision animal husbandry, including livestock health breeding environment and intelligent equipment, air quality and biosecurity engineering for livestock farms, and epidemic prevention measures. She has extensive experience in intelligent diagnosis models and systems for typical livestock and poultry diseases, as well as intelligent epidemic disease early warning. She also contributed to the development and application of innovative pollutant real-time measurement techniques, especially for the odor concentration in livestock and poultry farms.

**Peiguang Xin** is currently a Ph.D. candidate in Agricultural Bio-environment and Energy Engineering at the College of Water Resources and Civil Engineering, China Agricultural University, with long-term research focusing on multimodal intelligent perception and health monitoring of livestock and poultry. The research centers on precision management and early disease warning of growing–finishing pigs. The author has participated in several research projects, including a subproject of the national “Science and Technology Innovation 2030—New Generation Artificial Intelligence” Major Program and a key R&D project of the Ningxia Hui Autonomous Region, taking responsibility for multimodal data acquisition, core algorithm design, and prototype system development. The main research interests include joint weight–posture modeling, intelligent weight estimation under multi-posture conditions, and pen-wise group matching-based individual identification. The author has published one high-level journal paper on livestock and poultry sound detection and has applied for two Chinese invention patents related to group-housed pig weight estimation methods.

**Qin Tong** is an Associate Professor at the Department of Agricultural Buildings and Environmental Engineering, College of Water Resources and Civil Engineering. PhD from the Royal Veterinary College, University of London, and recipient of the Marie Curie Young Researcher Fellowship. Participated in two EU Seventh Framework Program projects. Mainly engaged in teaching and research on precision livestock farming, animal behavior and welfare. Principal investigator of two projects funded by the National Natural Science Foundation, and four subprojects under the National Key R&D Program, including one key project of China-UK AgriTech Challenge. Conducted extensive innovative research in lighting incubation of poultry, inspection and spray immunization robotic equipment in poultry house, and published over 40 research papers.

**Chao Liang** is an Associate Professor at the College of Water Resources and Civil Engineering, China Agricultural University. He is a recipient of the National Postdoctoral Innovative Talent Support Program, and serves as a Council Member of the Animal Husbandry Engineering Branch of the Chinese Society of Agricultural Engineering and a Youth Editorial Board Member of the journal Building Simulation. His long-term research and teaching focus on precision control of livestock and poultry breeding environments, energy conservation and carbon reduction in livestock housing, and smart farming and robotics. Over the past five years, he has led or participated in over 10 research projects, and has published more than 20 academic papers as first or corresponding author in top journals and has applied for/been granted over 10 patents.

**Ligen Yu** earned a Ph.D. in Agricultural Biological Environment and Energy Engineering from the China Agricultural University in 2013. He has worked for the Information Technology Research Center, Beijing Academy of Agriculture and Forestry Sciences, and since 2018 has been an Associate Researcher in the National Engineering Research Center for Agricultural Information Technology. Currently, he serves as the Deputy Director of the Department of Animal Husbandry and Information‌. Ligen Yu has extensive experience in animal audio perception recognition, intelligent disease diagnosis and early warning technology for livestock and poultry. He has also contributed to the development and application of innovative farming technology for animals.

**Aiqiao Liu** earned a postgraduate degree in 2009. She has been deeply engaged in the agriculture and animal husbandry industry for over three decades, consistently focusing on innovation in agricultural science and technology. By leveraging biotechnology to strengthen the industrial foundation, she has led the team in successfully breeding the “Jinghong, Jingfen, and Jingbai” laying hens and the “Wode series” broilers, contributing to a leap forward in China poultry breeding industry. Driving industrial transformation through digital intelligence technology, she pioneered the smart layer “industrial internet platform.” By deeply integrating information technology with biotechnology, she has established a new digital economy ecosystem for the poultry industry, providing comprehensive solutions for its digital and intelligent transformation and promoting high-quality development of the sector.

**Chaoyuan Wang** is a full professor of the Department of Agricultural Structure and Bioenvironmental Engineering, College of Water Resources and Civil Engineering, China Agricultural University. He is currently serving the deputy director of the Key Laboratory of Agricultural Engineering in Structure and Environment, Ministry of Agricultural and Rural Affairs of China, and chairman of the Animal Husbandry Engineering Branch of the Chinese Society of Agricultural Engineering (CSAE). Dr Wang’s research interests include animal housing and environment control, air quality and energy management in animal production, intelligent sensing of animal behavior and physiology, as well as smart diagnostics and early warning systems for animal diseases. He has published over 140 journal articles and 60 conference papers/abstracts, contributed to the editing of 20 books and chapters.
